# Editorial: Cephalopod Research Across Scales - Molecules to Ecosystems

**DOI:** 10.3389/fphys.2021.752075

**Published:** 2021-10-04

**Authors:** Erica A. G. Vidal, Rui Rosa, Graziano Fiorito

**Affiliations:** ^1^Center for Marine Studies – Federal University of Parana (UFPR), Pontal do Paraná, Brazil; ^2^MARE – Centro de Ciências do Mar e do Ambiente, Laboratório Marítimo da Guia, Faculdade de Ciências da Universidade de Lisboa, Lisbon, Portugal; ^3^Department of Biology and Evolution of Marine Organisms, Stazione Zoologica Anton Dohrn, Naples, Italy

**Keywords:** behavior, cephalopods, muscle physiology and contractility, ocean acidification, paralarvae, reproductive tactics, sexual selection, tentacles

This Research Topic aims to draw a picture on recent advances in cephalopod research inspired by selected papers from the Cephalopod International Advisory Council Conference—CIAC 2018 held in Saint Petersburg, Florida, USA, during 12–16 November, 2018, but also includes papers on topics encompassing the theme of the conference, “*Cephalopod Research Across Scales - Molecules to Ecosystems*.” Along with this Research Topic there are two other companion special issues in Bulletin of Marine Science (Judkins et al., 2020) and in Fisheries Research (González and Pierce, 2021) that include papers from the CIAC 2018 Conference. The conference was preceded by five workshops lasting over two days (10 and 11 November) on themes of particular interest by the scientific community, as a tradition during CIAC conferences and a brief overview of the goals and outcomes of the workshops are also introduced here. Other accounts of the CIAC 2018 Conference and workshops can be found at the CIAC web site (https://cephalopod.wordpress.com).

Overall, this Research Topic consists of 12 contributions. Nine of the papers are primary research articles, one of them is a hypothesis theory driven paper and, there are two review articles. The overarching topics that are being addressed by the contributions in this Research Topic reflect several key issues that provide a comprehensive and integrative view on cephalopod physiology and related subthemes, including sexual selection, behavior, climate change, feeding and metabolism, and welfare.

Intra-sexual selection includes processes such as sperm competition and cryptic female choice, and one of the consequences of intra-sexual selection is that male reproductive traits tend to evolve and diverge at high rates. Ibáñez et al. tested this hypothesis by studying the evolution and diversification of several reproductive traits (e.g., hectocotylized arm length, ligula, and spermatophore lengths) across benthic octopuses (including 87 species), employing phylogenetic analytical methods. The results point out that male reproductive traits have evolved in a correlated way with body size and exhibited accelerated rates of evolution (at least in several Antarctic and deep-water lineages) presumably due to sexual selection. Morse and Huffard presented a riveted and comprehensive review summarizing the current knowledge of reproductive behavior within the Cephalopoda. The authors have combined existing information on pre- and post-copulatory behaviors to build novel insights, relating to cephalopod mate choice, sensory ecology, and the proliferation of polyandry among the class. They have concluded that sperm competition and possibly cryptic female choice are likely to be critical determinants of which individuals' alleles get transferred to subsequent generations in cephalopod mating systems. Gaps within the current knowledge of how sexual selection operates are also uncovered and highlighted, valuably indicating areas of interest for new research on the elaborated mating systems of cephalopods.

Further exploring the sexual selection topic, but addressing the perspective of squids, Marian et al. provide a rich review on male alternative reproductive tactics in loliginids. The authors argue that loliginid squid provide unique models to explore sexual selection as they have two distinct fertilization environments in the female body that differ in aspects such as fertilization timing and success and, are associated with male alternative reproductive tactics, namely “sneaker” and “consort.” Large consort males fight other males to gain access to females and deposit spermatophores within the female mantle cavity, while small sneaker males, engage in furtive mating and deposit spermatophores near the female buccal seminal receptacle. These results lead to the conclusion that several ejaculate traits (e.g., spermatophore and sperm morphology and functioning) have unique features that may have evolved in response to the fertilization environment faced by each temporary or permanent male morph.

Apostólico and Marian contribution deals with an unexplored sexual selection topic in cephalopods: the underlying mechanisms responsible for the expression of male alternative reproductive phenotypes. Based on observations made in captivity they described, first-time, males of the loliginid squid *Dorytheuthis pleii* of intermediate size and age displaying behaviors of both sneakers and consorts males simultaneously, leading to the hypothesis of them being a transitional stage between these both phenotypes. The result corroborates the ontogenetic hypothesis, indicating that small young males adopt sneaker tactics to mate instead of competing with large consort males for females, but as they grow, they modify the morphology of their ejaculates and mating behavior, going through an “intermediate” stage, before becoming large consort males.

Evaluation of the ontogenetic underlying factors responsible for the expression of a particular behavior provide valuable insights into developmental constraints (Boletzky, 1997) and ultimately, the way behavioral adaptations have evolved. Vidal and Salvador documented the ontogeny of predatory behaviors in *Doryteuthis opalescens* looking closely at the specialized tentacular strike behavior of squids. Loliginid paralarvae do not capture prey as adults, they use progressive predatory behaviors in which the tentacles function as arms, because important morphological and structural features are not yet formed, namely, clubs, stalks, and muscle fibers. By examining the relationship between overall morphology and predatory behaviors, the authors were able to uncover the interconnected morphological and behavior traits that enabled squid to perform the tentacular strike behavior (i.e., clubs, stalks and arm crown development, and schooling behavior), leading to the conclusion that the expression of the tentacular strike involves different levels of development and thus represents a major developmental milestone.

Cephalopods are highly diverse and thrive in a wide range of marine habitats, from coastal waters to the deep-sea, reflecting their successful adaptations to distinct habitats and motivate the need to learn more about the interrelationships between their life-cycles and environmental conditions (Rodhouse et al., 2014; Schickele et al., 2021). One of the major threats to life in the oceans is ocean acidification (OA). Presently, there is an increasing evidence of detrimental OA effects on the behavioral ecology of certain marine taxa, including cephalopods (e.g., Gutowska et al., 2010). Of particular interest are the sensitivity of cephalopod early life stages—particularly embryos and paralarvae—to environmental stressors related to OA, and two of the papers in this Research Topic have addressed this issue. By studying the developmental and behavioral ecology (namely shelter-seeking, hunting, and response to a visual alarm cue) of the common cuttlefish, *Sepia officinalis* early life stages, Moura et al. argue that the degree of phenotypic plasticity shown by the early ontogenetic stages to OA is linked to: (i) the great adaptability of cuttlefish to highly dynamic coastal and estuarine zones, and (ii) the fact that the embryos already face harsh conditions (hypoxia and hypercapnia) inside their eggs during early development. Zakroff and Mooney report the results of their experimental study exposing *Doryteuthis pealeii* embryos and paralarvae to the combined effects of OA and warming. They found that when reared under severe, chronic acidification and warming these early stages show a range of responses from sensitive to resistant. For example, time to hatching, which increased with acidification, decreased drastically under warming and, further, removed delays caused by acidification driven by between clutch differences. Noticeably, their study strongly aligns with the theme of this Research Topic, showing how global processes (OA and warming) interact across biological (individual, egg capsule, and clutch) and temporal (across the breeding season) scales to affect squid development.

Abiotic conditions are known to affect processes of growth and sexual development, and biochemical composition of cephalopods. Pascual et al. showed significant changes in biochemical composition (e.g., protein, glucose, cholesterol, acylglycerides, glycogen) of the gonad, hepatopancreas and muscle of *Octopus maya* relative to sex and season. Such findings may serve as reference values for several physiological indicators in *O. maya*, useful for programs monitoring wild populations, as well as to design diets and management protocols to produce octopus under controlled conditions. Regarding cephalopod growth patterns, it is known that young juvenile *Sepia officinalis* can grow up to 12% body weight per day, but how the metabolic costs associated with such substantial growth rate impacts muscle performance is unknown. Lamarre et al. integrated several aspects of contractility, protein synthesis, and energy metabolism in mantle of juvenile cuttlefish to provide a comprehensive insight into these biochemical processes.

Considering that cephalopods high metabolism is fueled by a protein-based diet in a muscular body, muscle physiology studies (e.g., Kier, 2016) may bring into the light important facts to improve our understanding of the adaptability of cephalopods to environmental conditions. One major pathway involved in muscle metabolism is the evolutionary conserved mTOR-signaling cascade, which regulates cell growth and homeostasis in response to a wide range of factors. Maiole et al. tackle this topic by characterizing *Octopus vulgaris* mTOR functional domains through designing and testing an *in vitro* protocol of resistance exercise and inducing fatigue in arm samples. They found a high level of homology with vertebrates' mTOR and revealed the activation of the mTORC1 pathway in the exercise paradigm, which suggests that mTORC1 activity can be used as a marker to assess cephalopod response to changes in physiological and ecological conditions and, thus improve their maintenance and welfare.

Cephalopod welfare has become a timely topic that attracts great interest recently (Fiorito et al., 2015) due to the increasing importance of cephalopods in scientific and commercial activities requiring a clear understanding of their physiological and behavioral responses to anesthetic agents. Roumbedakis et al., hit the target with a study that evaluated short and long-term effects of anesthesia using different agents (magnesium chloride, ethanol, and clove oil) and cold seawater (11 and 13°C) on growth and mortality of *Octopus maya* juveniles. Based on the adopted criteria, metabolic (oxygen consumption) and behavioral (prey capture) responses to anesthesia were evaluated. Exposure to all concentrations tested of magnesium chloride, cold seawater (13°C), and clove oil reduced or inhibited the incidence of attacks to prey after recovery from anesthesia. Only clove oil proved to have a long-term effect on growth. Based on the results, the authors propose that anesthesia can be suppressed during short-term handling (<180 s), when no pain, distress, or suffering are involved. But when handling requires an anesthetized animal, the use of ethanol (3.0%) or cold sea water (11°C) were recommended to improve octopus welfare.

Processes of vomiting or regurgitation have been described in several invertebrate marine species, which prompt consideration of whether cephalopods have also this capability. In a stimulating “hypothesis and theory” article, Sykes et al. provide several different rationales to support the idea that cephalopods can vomit. Among the different lines of thought, the authors describe anecdotal reports of regurgitation-like behavior in several species, including *Sepia officinalis, Sepioteuthis sepioidea, O. vulgaris*, and *Enteroctopus dofleini*.

In compiling this Research Topic, we hope to provide an overview toward timely, significant and innovative cephalopod research, while also framing areas for future work, usefully highlighted in several of the papers shared here, to encourage wide readership in this exciting field of research. Our special thanks go to the 56 authors, representing 13 countries and, also to the reviewers for their valuable assistance. Finally, we thank the chair of the CIAC 2018, Heather Judkins. CIAC conferences are the opportune and stimulating occasion for outstanding scientific content encouraged by a warm atmosphere! We look forward for the next CIAC conference that will be held during 2–8 April, 2022 in Sesimbra, Portugal, with the theme “*Cephalopods in the Anthropocene: multiple challenges in a changing ocean*.”

## Workshops

The workshop “*Paralarval and juvenile cephalopods: an updated identification guide*,” led by Erica A. G. Vidal, Liz Shea, and Heather Judkins focused on disseminating knowledge on the identification of cephalopod early life stages and on gathering a team of researchers to compile existing taxonomic information to create an updated book for the identification of cephalopod early life stages. During the workshop, the 27 participants from 13 countries, adopted a “hands on” approach and worked in groups on identifying cephalopod early life stages, sharing expertise on particular families and creating an effective interaction atmosphere between students and experts in the field. The output from this workshop will be the book “*Cephalopod Early-life stages: An Identification Handbook*” that is being prepared to be published by Springer Nature in 2022.

The workshop “*The biogeochemical role of cephalopods in the world's oceans*,” led by Henk-Jan Hoving, aimed to gather the available knowledge on the role of cephalopods in the oceans' biogeochemistry and energy transfer (e.g., migration, consumption, respiration and excretion, terminal spawning) in marine food webs, with the ultimate goal to provide a comprehensive and integrative review paper on the topic.

The workshop “*Hard structures of cephalopods and their application in your field of study*” was led by Alexander Arkhipkin, Catalina Perales-Raya, and Fedor Lishchenko, with the main goal to have the most recent update on use of recording structures in studies of cephalopod taxonomy, age, growth, and population structure. The workshop was attended by 23 participants (scientists as well as students) from eight countries around the Globe. Theoretical sessions included general introduction on the recording structures of cephalopods, specific methodologies dedicated to processing of statoliths, cephalopod beaks, and gladii, as well as their shape analysis. Additionally, Kathleen Ritterbush gave a talk on the shell hydrodynamics of ammonoid cephalopods. Practical sessions took place with participants having first-hand experience in extraction of recording structures (statoliths, beaks, and stylets of octopuses) from cephalopod bodies, their sectioning and examination. At the final part of the workshop a broad discussion on the key knowledge gaps and promising fields for future studies took place. The most important subjects for future studies included (a) Terminology-related issues; (b) Physiology of increment deposition; (c) Problems with aging accuracy and precision; (d) Validation methods and associated problems; (e) The best aging methods for each group of species; (f) Species in which hard structures provide reliable tools for age estimation and, (g) Species for future research.

The workshop: “*Genetic Tools and Live Imaging in Cephalopods*” led by Eric Edsinger had a hands on approach with computer software interaction to cover several practical issues on implementing genetic and live imaging tools for cephalopods, namely: (1) Identifying genes of interest in annotated genomes, (2) Designing transgenic reporters, biosensors, and CRISPR-Cas9 guide RNAs, (3) Injection of mRNA/constructs/CRISPR-Cas9, (4) Live imaging approaches for light-based genetic tools and, (5) Applications of genomic resources and genetic tools to cephalopod biology, emerging genetic models, among others future prospects. During the workshop, embryos, and hatchlings were produced and used for expressing injected mRNAs, transgenic constructs, or genome edited genes, allowing a practical, interactive and also first-hand experience among the participants.

The workshop “*Cephalopod Science: the direction of future research and the relevance of new policies*” led by Giovanna Ponte, Ian Gleadall, and Graziano Fiorito provide the ground for a brainstorming session to identify likely avenues for novel ground-breaking cephalopod research areas and their potential effects on and benefits to human society. A discussion on the changes in policy for experimentation and fisheries of cephalopods occurring in different regions of the world and the potential effects of these changes on cephalopod research in both global and local contexts were also addressed, with the aim to prepare a white paper to summarize and report on these topics.

## Author Contributions

All authors listed have made a substantial, direct and intellectual contribution to the work, and approved it for publication.

## Conflict of Interest

The authors declare that the research was conducted in the absence of any commercial or financial relationships that could be construed as a potential conflict of interest.

## Publisher's Note

All claims expressed in this article are solely those of the authors and do not necessarily represent those of their affiliated organizations, or those of the publisher, the editors and the reviewers. Any product that may be evaluated in this article, or claim that may be made by its manufacturer, is not guaranteed or endorsed by the publisher.
